# Genome Sequence of Hydrocarbon-Degrading *Cronobacter* sp. Strain DJ34 Isolated from Crude Oil-Containing Sludge from the Duliajan Oil Fields, Assam, India

**DOI:** 10.1128/genomeA.01321-15

**Published:** 2015-11-12

**Authors:** Siddhartha Pal, Tirtha Das Banerjee, Ajoy Roy, Pinaki Sar, Sufia K. Kazy

**Affiliations:** a Department of Biotechnology, National Institute of Technology Durgapur, Durgapur, India; b Department of Biotechnology, Indian Institute of Technology Kharagpur, Kharagpur, India

## Abstract

We report here the 4,856,096-bp draft genome sequence of hydrocarbon-degrading *Cronobacter* sp. strain DJ34 isolated from crude oil-containing sludge from the Duliajan oil fields, India. DJ34 contains genes that mediate hydrocarbon degradation, metal resistance, and biosurfactant production. This is the first report of the genome sequence of *Cronobacter* sp. inhabiting an oil-contaminated environment.

## GENOME ANNOUNCEMENT

Genome sequences of *Cronobacter* spp., commonly described as opportunistic food-borne pathogens causing fatal neonatal diseases, have already been reported (1, 2). To date, no information is available on the genome sequences of members of this genus that inhabit petroleum oil-rich environments. Very few strains of *Cronobacter* spp. have been reported from hydrocarbon- or industrial waste-contaminated habitats (3, 4). Recently, we isolated a Gram-negative, facultative anaerobic, hydrocarbon-degrading strain, *Cronobacter* sp. DJ34, from crude oil-containing sludge in the Duliajan oil fields, Assam, India. The 16S rRNA gene sequence of the isolate has been deposited in NCBI GenBank under the accession no. KM054665, which showed 99% sequence similarity with *Cronobacter pulveris* strain E444 (accession no. EF059835). Strain DJ34 showed multiple heavy metal resistances, growth under a wide range of pH, temperature, and salinity conditions, biosurfactant production, and an ability to utilize various electron acceptors during anaerobic growth. The genome sequence of this bacterium was determined to obtain better insights into the metabolic versatility of the strain. To our knowledge, this is the first report on the genome sequence of a *Cronobacter* sp. inhabiting an oil-contaminated environment.

The genomic DNA of DJ34 was extracted using the Promega Wizard genomic DNA purification kit (Promega, USA), according to the manufacturer’s protocol. The draft genome sequence was obtained by using the Illumina HiSeq 2500 platform. The shotgun sequencing generated 5,092,150 high-quality paired-end reads with 100-fold coverage. Filtered reads were assembled by using *de novo* assemblers A5 (version 20140113) (5), ABySS (version 1.3.7) (6), MaSuRCa (version 2.3.2) (7), and SPAdes (version 3.1.1) (8). Integration of contigs was done with CISA (version 1.3). A total of 30 contigs with *N*_50_ length of 314,448 bp and average length of 161,869 bp were assembled. Genome annotation was performed by NCBI Prokaryotic Genomes Automatic Annotation Pipeline using Glimmer, tRNAscan-SE, and RNAmmer tools. Functional annotation was carried out using the RAST server with the SEED database (9).

The draft genome is composed of 4,856,096 bp, with a G+C content of 56.5%. A total of 4,382 coding sequences (CDSs), 60 pseudogenes, and 14 noncoding RNA (ncRNA), 76 tRNA, and 23 rRNA genes were identified. The CDSs included many genes previously described for other *Cronobacter* spp. such as genes for secretion systems, superoxide dismutase, hemolysin, O antigen, enterobactin, biofilm formation, metalloprotease, adhesins, antibiotic resistance, heavy metal resistance, iron acquisition, universal stress proteins (*uspA* and *uspB*), starvation-sensing protein (*rspA*), flagella, fimbriae, phages, etc. (10). The genome of *Cronobacter* sp. DJ34 also contained different hydrocarbon degradation-related genes, including alkane-1-monooxygenase, alkane sulfonate monooxygenase, benzoylformate decarboxylase, 4-carboxymuconolactone decarboxylase, 3-carboxy-*cis*,*cis*-muconate cycloisomerase, 3-oxoadipate-enol-lactonase, 3-oxoadipate coenzyme A (CoA)-transferase, *bph*, protocatechuate 3,4 dioxygenase, salicylaldehyde dehydrogenase, etc. In addition to these, dissimilatory nitrate, nitrite and sulfite reductase, and biosurfactant synthesis genes have been noted. A detailed genome sequence analysis may provide deep insights into the genetic and metabolic potential of this organism for hydrocarbon degradation and survival in an oil-contaminated habitat.

### Nucleotide sequence accession numbers.

This whole-genome shotgun project of *Cronobacter* sp. DJ34 has been deposited at DDBJ/EMBL/GenBank under the accession no. LFEJ00000000**.** The version described here is the first version, LFEJ01000000.
